# Protective Effects of Carbon Monoxide-Releasing Molecule-2 on the Barrier Function of Intestinal Epithelial Cells

**DOI:** 10.1371/journal.pone.0104032

**Published:** 2014-08-07

**Authors:** Xinwei Mu, Chen Pan, Shuyun Zheng, Yasir Alhamdi, Bingwei Sun, Qiankun Shi, Xiang Wang, Zhiwei Sun, Chenghock Toh, Guozheng Wang

**Affiliations:** 1 Department of Critical Care Medicine, Nanjing First Hospital, Nanjing Medical University, Nanjiang, Jiangsu Province, PR China; 2 Department of Critical Care Medicine, Nanjing Children^,^s Hospital, Nanjing Medical University, Nanjiang, Jiangsu Province, PR China; 3 Institute of Infection and Global Health, the University of Liverpool, Liverpool, United Kingdom; 4 Department of Burn and Plastic Surgery, Affiliated Hospital, Jiangsu University, Zhenjiang, Jiangsu Province, PR China; Emory University School of Medicine, United States of America

## Abstract

**Objective:**

To investigate the protective effects and mechanisms of carbon monoxide-releasing molecule-2 (CORM-2) on barrier function of intestinal epithelial cells.

**Materials and Methods:**

After pre-incubation with CORM-2 for 1 hour, cultured intestinal epithelial IEC-6 cells were stimulated with 50 µg/ml lipopolysaccharides (LPS). Cytokines levels in culture medium were detected using ELISA kits. Trans-epithelial electrical resistance (TER) of IEC-6 cell monolayers in Transwells were measured with a Millipore electric resistance system (ERS-2; Millipore) and calculated as Ω/cm^2^ at different time points after LPS treatment. The permeability changes were also measured using FITC-dextran. The levels of tight junction (TJ) proteins (occludin and ZO-1) and myosin light chain (MLC) phosphorylation were detected using Western blotting with specific antibodies. The subsequent structural changes of TJ were visualized using transmission electron microscopy (TEM).

**Results:**

CORM-2 significantly reduced LPS-induced secretion of TNF-α and IL-1β. The LPS-induced decrease of TER and increase of permeability to FITC-dextran were inhibited by CORM-2 in a concentration dependent manner (P<0.05). LPS-induced reduction of tight junction proteins and increase of MLC phosphorylation were also attenuated. In LPS-treated cells, TEM showed diminished electron-dense material and interruption of TJ and desmosomes between the apical lateral margins of adjoining cells, which were prevented by CORM-2 treatment.

**Conclusions:**

The present study demonstrates that CORM-2, as a novel CO-releasing molecule, has ability to protect the barrier function of LPS-stimulated intestinal epithelial cells. Inhibition of inflammatory cytokines release, restoration of TJ proteins and suppression of MLC phosphorylation are among the protective effects of CORM-2.

## Introduction

Carbon monoxide (CO) was considered as a toxic gas for a long time due to its higher affinity for hemoglobin than oxygen. In recent years, some researchers have found that CO has additional functions in biological systems than was first thought. In mammalian cells, a small amount of endogenous CO is produced by heme oxygenase-1 (HO-1), a stress-inducible protein that catalyses conversion of heme into free Fe^2+^, CO, and biliverdin [1], [2]. An elevated level of CO was detected in exhaled air of critically ill patients as a result of HO-1 induction, which was considered to be a self-protective mechanism of an organism [3]. It has also been reported that induction of HO-1 or exogenous CO administration could improve the survival of septic mice [4], [5]. In a rat model of hepatic ischemia-reperfusion injury (I/Ri), exogenous CO attenuated the liver damage caused by I/Ri [6]. Other biological functions of CO include anti-inflammation, anti-thrombosis and vasodilatation [7]–[10]. Therefore, increasing attention has been focused on this diatomic gas as a potential messenger.

However, it is not easy to conduct experiments with CO, due to the formation of carboxyhemoglobin. Carbon monoxide-releasing molecules (CO-RMs) are a class of newly synthesized transition metal carbonyls which can release CO in a controlled manner without significantly altering the level of carboxyhemoglobin in animal models. CORM-2 is one of this type of compounds, which releases CO when it is dissolved in dimethyl sulfoxide (DMSO). In fact, CORM-2 has been widely used as a CO donor in a variety of experiments. In nerve-injured mice, CORM-2 administration reduced the neuropathic pain [11]. CROM-2 could also attenuate leukocytes infiltration in the renal tissue of thermally injured mice [12]. Moreover, CORM-2 could inhibit the migration of osteoarthritic synoviocytes [13].

Intestinal epithelial cells serve as the first line of defense against pathogens in the intestinal tract, playing an important role in maintenance of the gut barrier function. When permeability of intestinal epithelial cells increases, pathogens in the lumen will enter the systemic circulation, and subsequently contribute to the development of systemic inflammation. Kazuhiro Katada et al. showed that systemic administration of CORM-2 in mice attenuated ischemia/reperfusion-induced inflammation in the small intestine [14]. Moreover, other researchers reported that CORM-2 could suppress the inflammatory response induced by cytokines in Caco-2 cells, a human intestinal carcinoma cell line [15]. Thus, we hypothesized that CORM-2 may provide protective effects for the intestine when inflammation occurred. To our knowledge, there little is known about the effects of CO on the function of the intestinal barrier function. In this work, we chose IEC-6 cells, non-transformed intestinal epithelial cells, to form the cell monolayer. IEC-6 cells originally from the small intestinal crypt cells of the rat maintain the features of normal intestinal cells, such as tight junctions linking adjacent cells [16]. LPS, a component of the wall of Gram-negative bacteria, has been identified to possess the ability of interrupting the intestinal barrier [17] and has been used to mimic the conditions of inflammation and test therapeutic reagents [18].

The aim of this study is to examine whether CORM-2 can protect the barrier function of intestinal cell monolayer against inflammation. Furthermore, we have explored the possible mechanisms underlying this phenomenon. Our data indicate that COMR-2 can enhance the barrier function of the IEC-6 cell monolayer and CORM-2-derived CO is the effective factor. In addition, pro-inflammatory cytokines, TJ proteins, MLC phosphorylation, are all involved in the process.

## Materials and Methods

### Chemicals and Reagents

Anti-ZO-1, anti-β-actin (Santa Cruz Biotechnology), anti-phospho-myosin light chain (MLC) and anti-total-MLC (Cell Signaling), anti-occludin (Abcam) antibodies and peroxidase-conjugated goat anti-rabbit secondary antibody (Jackson Immunologicals) were purchased from relevant manufacturers. CORM-2 and LPS (*Escherichia coli* 055:B5) were purchased from Sigma-Aldrich. CORM-2 was dissolved in DMSO and then diluted in culture media to achieve the required concentrations. Inactivated CORM-2 (iCORM-2) was prepared by incubating CORM-2 for 24 h at 37°C to liberate all CO.

### Cell culture

IEC-6 cells were purchased from the American Type Culture Collection (ATCC, Manassas, VA) and maintained in Dulbecco’s modified Eagle’s medium with high glucose (DMEM, GibcoBRL) supplemented with 10% fetal bovine serum (FBS, GibcoBRL) and 10 µg/ml insulin (Sigma-Aldrich). Cells were cultured at 37°C in a humidified atmosphere with 5% CO_2_. Cells were used at the 15th to 25th passage for all experiments.

### Cytotoxicity assay

IEC-6 cells were seeded in 96-well culture plates at a concentration of 5×10^5^ cells/ml 24 h prior to experiments. After different treatments, the cells were washed 3 times with phosphate-buffered saline (PBS). Ten µl of 5 mg/ml 3-(4, 5-dimethylthiazol-2-yl) -2,5 diphenyltetrazolium bromide (MTT) was added to each well and the contents were incubated for 4 h at 37°C. The media was removed and the formazan crystals inside the cells were dissolved in 200 µL of DMSO. The absorbance of each well was measured at 450 nm on a microplate reader.

### Determination of trans-epithelial electrical resistance (TER) and permeability of the cell monolayer

TER values of IEC-6 cell monolayer were measured with a Millipore electric resistance system (ERS-2; Millipore), and calculated as Ω/cm^2^. The cells were seeded on inserts (0.4 µM pore size; Millipore) in 24-well transwell chambers. TER recorded in unseeded transwell inserts was subtracted from all values. Measurements were not started until the value reached 50 Ω/cm^2^. Trans-epithelial permeability for macromolecular tracers was measured with FITC-labeled Dextran (FD-40, Sigma). The cells were seeded on the inserts (0.4 µM pore size; Millipore) in a 12-well transwell chamber. After CORM-2 treatment, cells were stimulated with LPS for 24 h. Then the media in the bottom well was replaced with 1.5 mL DMEM, whilst media in the upper well was replaced with 0.5 mL DMEM containing FITC-Dextran at 10 mg/mL. After 1 h incubation, the amount of dextran presented in the bottom well was measured with a microplate reader.

### Cytokine analysis

The culture medium of IEC-6 cells was collected,then centrifuged at 3000 rpm, for 10 min at 4°C. Cytokines levels in cell culture medium were measured using ELISA kits (TNF-α from R&D systems and IL-1β from RayBiotech), according to the manufacturer’s instructions. All standards and samples were run in duplicate.

### Western blotting

Proteins were extracted from cultured cells using RIPA buffer and their concentrations were determined using a Bradford protein assay kit (BCA kit, Pierce Biotechnology). Equivalent protein samples were resolved on SDS-PAGE gels, then the proteins were transferred onto PVDF membrane, which was then blocked and probed with antibodies for occludin (1∶250), ZO-1 (1∶1000), β-actin (1∶1000), MLC (1∶1000), p-MLC (1∶1000) overnight at 4°C followed by incubation with HRP-conjugated secondary antibody (1∶5000) for 1 h at room temperature. Blots were visualized using an enhanced chemiluminescent kit (Thermo Fisher Scientific).

### Transmission electron microscopy

Fully confluent cultured IEC-6 cells were washed and fixed with 4% (v/v) glutaraldehyde for 2 h and then post-fixed with 1% (w/v) osmium tetroxide. Thin sections were cut and stained with uranyl acetate and lead citrate. Images were taken with an H-600 (Hitachi, Japan) transmission electron microscope operated at 75 kV and images were captured digitally. Ultrastructural observations were made from multiple sites (10) of junctional complexes that were clearly identified. At least three images from each treatment group were analyzed by three people in a blinded fashion.

#### Data and Statistical Analysis

Data were presented as the mean±S.D and analyzed using the ANOVA test for comparisons between more than 2 groups. If a statistical significance was achieved, Student-Newman-Keuls test was used for comparison between 2 groups. Values of P<0.05 were considered statistically significant.

## Results

### Cytotoxic effects of CORM-2 on IEC-6 cells *in vitro*

**Figure 1:**
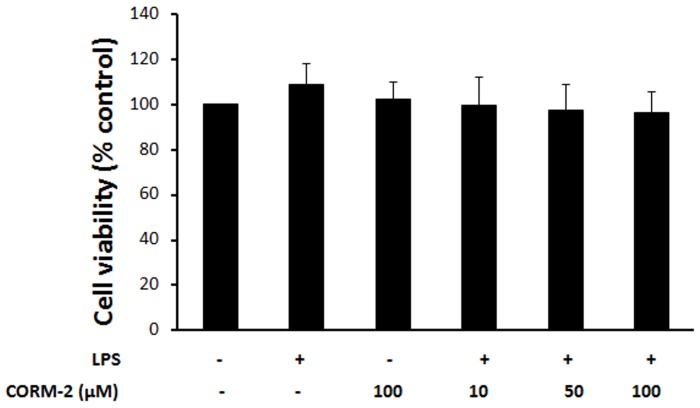
Effect of CORM-2 on cell viability. IEC-6 cells were treated with CORM-2 in the absence or presence of LPS (/ml) for 24 h, and cell viability was assessed using the MTT assay. Results are the mean h, and cell viability was assessed using the MTT assay. Results are the mean±SD of 3 independent experiments. ANOVA test P>0.05.

Previous studies have shown that CORM-2 is not toxic to airway epithelial cells, but it has not been applied to intestinal IEC-6 cells. To test if CORM-2 is toxic for IEC-6 cells, we treated the cells with CORM-2 for 24 h in the absence or presence of LPS and found that CORM-2 alone (up to 100 µM) or in combination with LPS (50 µg/ml) was not toxic to IEC-6 cells (Fig. 1).

### CORM-2 attenuates the reduction of barrier function in LPS-treated IEC-6 cells

**Figure 2:**
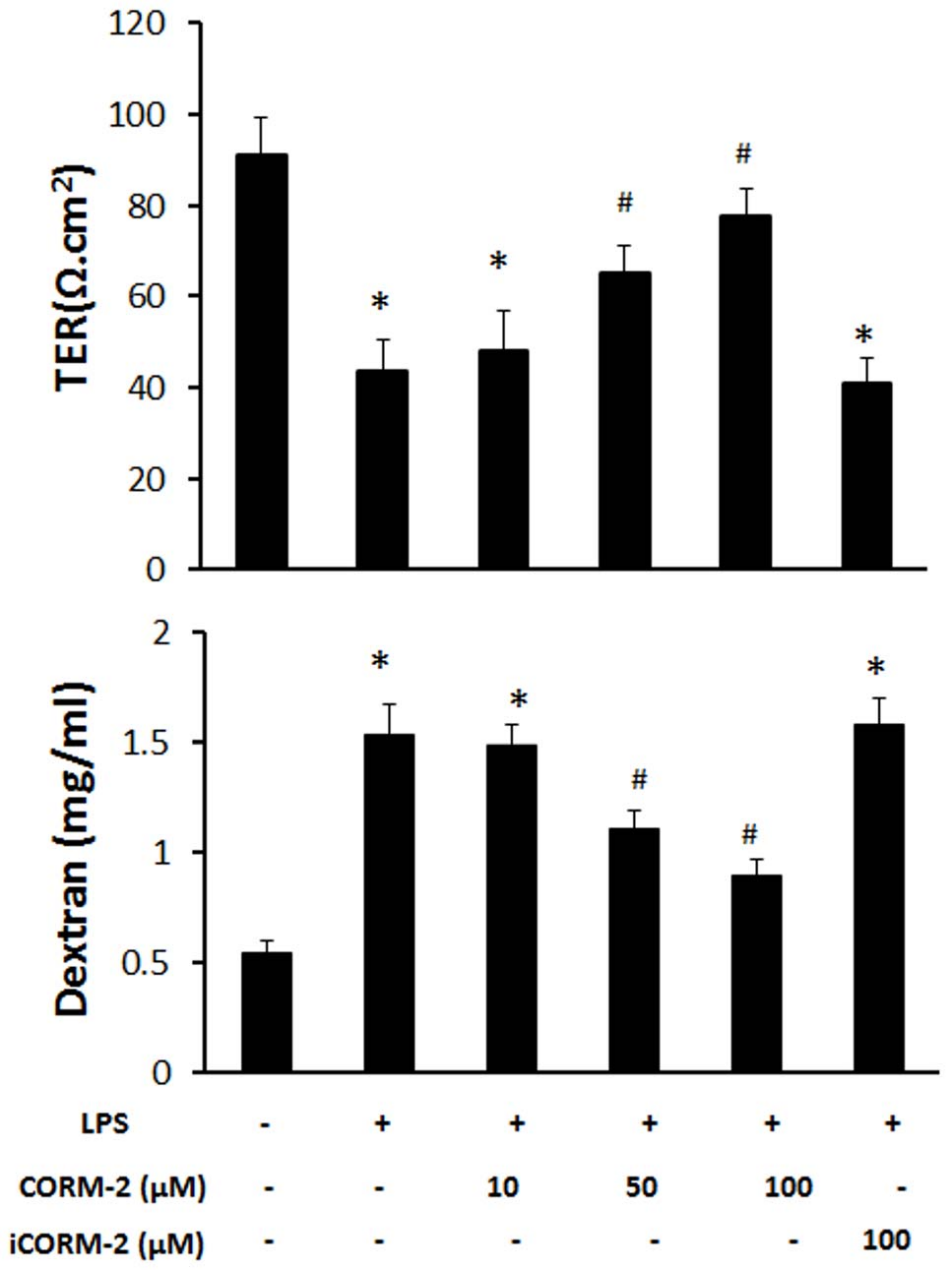
Effect of CORM-2 on the permeability of LPS-treated IEC-6 cell monolayer. When cells reached confluence in the transwell system, CORM-2 was added and incubated for 1 h. The cells were washed and treated with 50 h. The cells were washed and treated with 50 µg/ml LPS for 24 h. (a) Mean±SD of TER values from 3 independent experiments are shown. (b) Permeability of FITC-dextran across the cell monolayer indicated CORM-2 could decrease the LPS-induced increase. Results are the mean±SD of 3 independent experiments. *ANOVA test, *p*<0.05 as compared to control group, #*p*<0.05 as compared to LPS group.

To examine whether CORM-2 could protect the barrier function of the cell monolayer, ICE-6 cells were plated in a transwell system and TER values and FITC-dextran permeations were measured. LPS decreased the TER value from 91.2±8.2 Ω/cm^2^ to 43.6±7.1 Ω/cm^2^. Addition of CORM-2 attenuated this reduction in a concentration-dependent manner, but inactivated CORM-2 (iCORM-2) failed to produce this effect (Fig. 2a). FITC-dextran was then used as a probe to investigate the permeability of the IEC-6 cell monolayer. As shown in Fig. 2b, CORM-2 decreased the LPS-induced dextran leakage from the upper to the lower compartment in the transwell system, whilst iCORM-2 did not. These results suggest that CO released from CORM-2 is able to improve the barrier function of the IEC-6 cells (Fig. 2).

### CORM-2 inhibits release of TNF-α and IL-1β in LPS-treated IEC-6 cells

**Figure 3:**
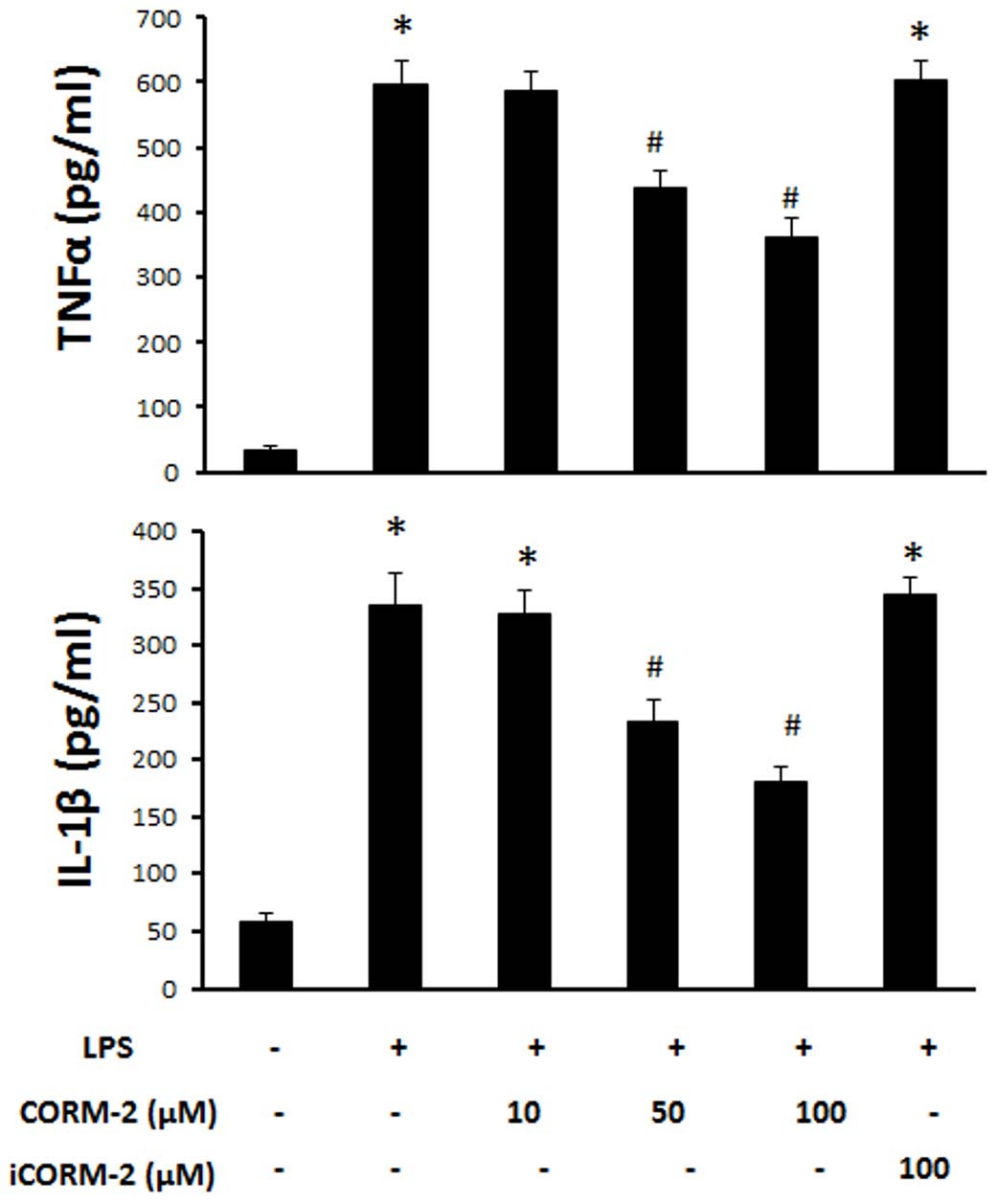
Effect of CORM-2 on the release of proinflammatory cytokines in LPS-treated IEC-6 cells. IEC-6 cells were pretreated with CORM-2 for 1 h and cells were washed and stimulated with 50 h and cells were washed and stimulated with 50 µg/ml LPS for 24 h. TNF h. TNF-**α** (a) and IL-1**β** (b) in the culture medium were measure using ELISA kits. Mean±SD from 3 independent experiments are shown. *ANOVA test, *P*<0.05 when compared to control group (no treatment), #ANOVA test, *P*<0.05 when compared to LPS alone group.

In order to explore the mechanism of COMR-2 protection of the barrier function of cell monolayer, we first investigated its effects on the release of proinflammatory cytokines from IEC-6 cells treated by LPS. Fig. 3a shows LPS significantly increased the release of TNF-α and addition of CORM-2 reduced the levels of TNF-α in the cell culture medium. Similarly, the increase in IL-1β was also attenuated by addition of CORM-2 whilst iCORM-2 showed no significant effect. This data indicates that CO released from CORM-2 can inhibit the secretion of LPS-induced proinflammatory cytokines (Fig. 3).

### *CORM-2* ameliorates down-regulation of TJ proteins in LPS-treated IEC-6 cells

**Figure 4:**
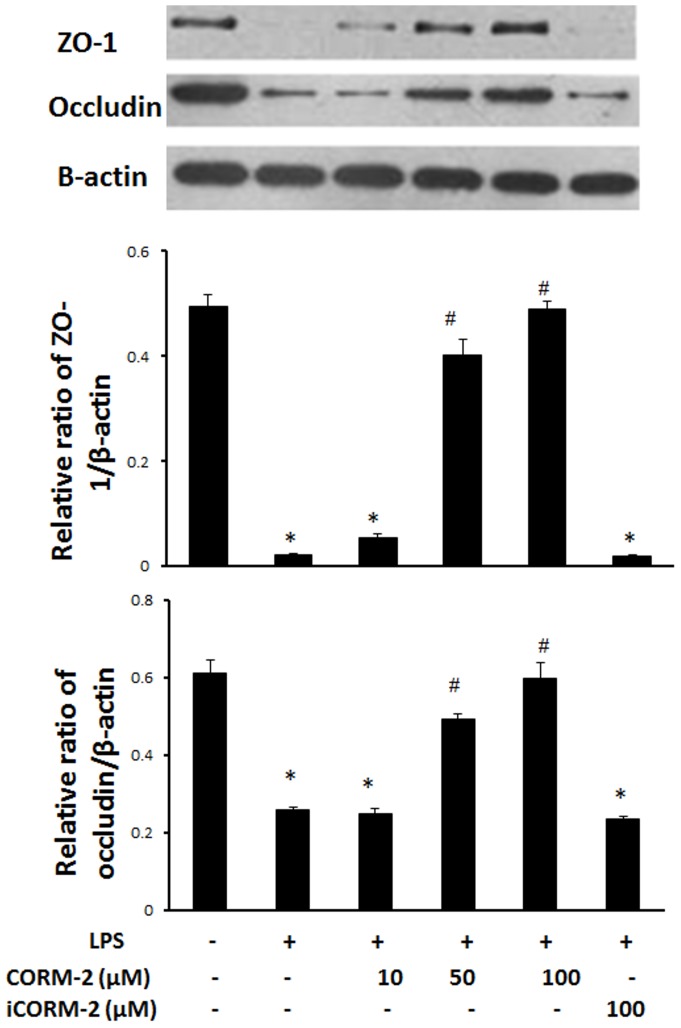
Effect of CORM-2 on TJ protein expression in LPS-treated IEC-6 cells. IEC-6 cells were pretreated with CORM-2 for 1 h and the cells were washed and stimulated with 50 h and the cells were washed and stimulated with 50 µg/ml LPS for 24 h. The cells were washed with PBS and harvested with RIPA buffer. The protein concentration was determined and proteins subjected to Western blotting (Methods). A typical image is shown (Upper panel). The relative ratios (Mean±SD) of ZO-1/β-actin (Middle panel) and occludin/β-actin (Lower panel) are calculated based on the densities of bands on Western blots from 3 independent experiments. *ANOVA test, *P*<0.05 when compared to control group (no treatment), #ANOVA test, *P*<0.05 when compared to LPS alone group.

We investigated whether the tight junction proteins, occludin and ZO-1, were involved in the protective mechanisms induced by CORM-2. Treatment of IEC-6 cells with LPS resulted in a significant reduction in the TJ proteins, occludin and ZO-1. Western blots showed that CORM-2, but not iCORM-2, increased the expression of ZO-1 and occludin (Fig. 4).

### CORM-2 suppresses myosin light chain (MLC) phosphorylation in LPS-treated IEC-6 cells

**Figure 5:**
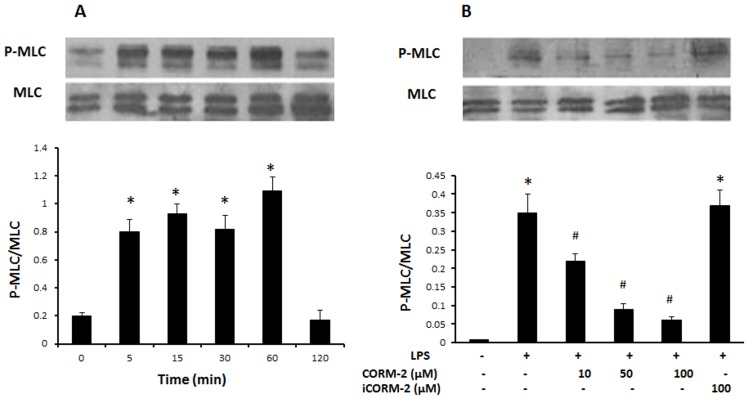
Effect of CORM-2 on the levels of MLC phosphorylation. (A) IEC-6 cells were treated with 50 µg/ml LPS and harvested at different time points. MLC phosphorylation (p-MLC) was detected using phosphorylation-specific antibody. Sample loading was normalized using anti-MLC antibody. A typical blot is shown (Upper panel). (B) IEC-6 cells were pretreated with CORM-2 for 1 h followed by LPS (50 µg/ml) treatment for 1 h. A typical Western blot shows that CORM h. A typical Western blot shows that CORM-2 reduced LPS-enhanced MLC phosphorylation (Upper panel). The relative ratios (Mean±SD) of p-MLC/MLC are calculated based on the densities of bands on Western blots from 3 independent experiments (Lower panels of (A) and (B)). *ANOVA test, *P*<0.05 when compared to control group (no treatment), #ANOVA test, *P*<0.05 when compared to LPS alone group.

To investigate further the mechanism of CORM-2 in protecting the barrier function of the cell monolayer, the level of myosin light chain (MLC) phosphorylation in IEC-6 cells was examined. Fig. 5a shows that the basal levels of phosphorylated MLC were low in untreated IEC-6 cells. Treatment with 50 µg/ml LPS caused an obvious increase in MLC phosphorylation. Pre-incubation of CORM-2 but not iCORM-2 with IEC-6 cells significantly inhibited LPS-induced the phosphorylation of MLC (Fig. 5).

### Changes of TJ in the transmission electron microscope

**Figure 6:**
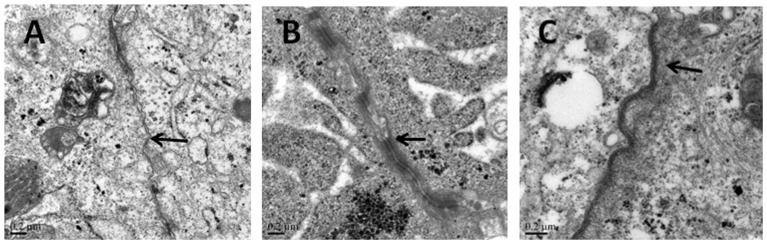
Changes of TJ observed by Transmission Electron Microscopy. Ultrastructure of TJs in a monolayer of cultured IEC-6 cells was observed by a transmission electron microscope. Panel (A) Normal control. (B) LPS–treated cells. The electron-dense materials were diminished in TJ and desmosomes. The space between adjoining cells was widened. (C) CORM-2 attenuated the LPS disruption of TJ and desmosomes. Arrows indicate the location of the TJs (Scale bar = 0.2 µm).

The ultrastructure of monolayers of IEC-6 cells was investigated by transmission electron microcopy (Fig. 6). The presence of electron-dense material in the intercellular space near the brush borders reflects the TJ. In cells without 50 µg/ml LPS treatment (control group Fig. 6a), the TJ and desmosomes displayed an intact structure. In cells treated with 50 µg/ml LPS (LPS-treated group Fig. 6b), the TJ and desmosomes were affected, this includes the loss of electron-dense material and the larger gap between adjoining cells. Conversely, pre-incubation with CORM-2 attenuated the LPS-induced disruption of TJ and desmosomes (Fig. 6c). These results demonstrate that CORM-2 reduced the distortion of the normal morphology of TJ complex induced by LPS.

## Discussion

The gastrointestinal tract functions not only as a digestive and absorptive organ, but also as a barrier against the pathogens in the lumen. A massive number of pathogenic bacteria colonize the gastrointestinal tract, and may cause systemic inflammation, such as sepsis, when they enter blood [19]. Sepsis, as a microvascular disease, may cause intestinal ischemia which further deteriorates the function of intestinal epithelial barrier [20], [21]. On the other hand, impaired barrier function promotes bacterial translocation and development of sepsis [22]. Therefore, it is crucial to protect the function of intestinal barrier in sepsis. In this study, we demonstrate for the first time that CORM-2 ameliorates the loss of barrier function of intestinal IEC-6 cells induced by LPS, by measuring the TER and permeability to a macromolecule, and the disruption to the TJ and desmosomes morphology observed under the TEM. Moreover, we observed that CORM-2 inhibits the secretion of proinflammatory cytokines. The TJ proteins, occludin and ZO-1, were restored by addition of CORM-2 and we also proved that CORM-2 could suppress the MLC phosphorylation in IEC-6 cells induced by LPS.

The effect of CORM-2 has also been investigated in Caco2 cells, a continuous cell line of heterogeneous human epithelial colorectal adenocarcinoma cells, and T84, a human colonic epithelial cell line [23], [24]. Both cell lines form polarized monolayer with much higher resistance tight junctions between adjacent cells and require much higher concentrations of LPS (800 µg/mL for Caco2 and 500 µg/mL for T84) to disrupt the barrier than that for IEC-6 cells (50 µg/mL) (Fig. S1 and S2). However, CORM-2 showed significant protective effect against high doses of LPS in term of permeability changes (Fig. S1 and S2). The TJ proteins, occludin, ZO-1 and claudin-1/4 in T84 cells, were restored by addition of CORM-2 (Fig. S3). These data have consolidated the findings with IEC-6 cells.

It is well known that HO-1 is not expressed under normal condition, but is up-regulated rapidly under various stimuli to enhance the production of endogenous CO, which is believed to be a defense mechanism for organisms [25]. It has been reported that induction of HO-1 can attenuate the damage of gastrointestinal mucosa [26]. Na Wang et al. have found that curcumin, a major active component of the food flavour turmeric, ameliorates hydrogen peroxide-induced barrier disruption of Caco-2 cells by up-regulating HO-1 [27]. Despite the fact that they identified that HO-1 was involved in the protective process, they did not investigate further which product of HO-1: Fe^2+^, CO, or biliverdin mediates this effect. In our study, we applied a CO-releasing molecule, CORM-2, to the monolayer of intestinal IEC-6 cells with inactivated CORM-2 (iCORM-2) as a control to demonstrate the effect of CO. The data obtained indicate that CO was the effective factor that protects the barrier function of intestinal epithelial cells. CORM-2 has been reported to have various effects on intestinal epithelial cells. Application of CORM-2 on duodenal mucosa in rats stimulates the release of HCO_3_^_^, which protects the duodenal mucosa against acid [28]. CO can also modulate colonic ion transporters, such as Na^+^-K^+^-2Cl^_^ cotransporter, Cl^_^/HCO3^_^ exchanger, and K^+^ channels [29]. Here, we found that CO ameliorates LPS-induced loss of barrier function of the intestinal epithelial cell monolayer *in vitro*. Thus, CO may play important roles in protecting the integrity of the intestinal mucosa epithelia *in vivo*.

Proinflammatory cytokines, such as TNF-α and IL-1β, contribute substantially to the loss of the function of intestinal epithelial barrier. Recent studies have shown that TNF-α and IL-1β directly increased the permeability of intestinal epithelial tight junction [30]–[32]. TNF-α and IL-1β, as early-stage proinflammatory cytokines, are usually released at the start of inflammation. In this study, we observed that the release of TNF-α and IL-1β by IEC-6 cells was increased dramatically after challenge with LPS. Although it remains unknown whether the barrier loss caused by LPS is derived mainly from the direct effect of LPS or from the LPS-induced cytokines, TNF-α and IL-1β, however, at least they can account partly for the barrier loss of IEC-6 cells. Moreover, we found that CORM-2 has the ability to inhibit release of TNF-α and IL-1β from intestinal cells. Therefore, the inhibition of proinflammatory cytokines by CORM-2 is probably involved in protection on barrier function. In addition, it has been also reported that TNF-α and IL-1β induced barrier dysfunction requires activation of the NF-κB pathway [30], [32]. NF-κB is a transcription factor which controls gene expression of various proinflammatory cytokines. LPS stimulation can markedly activate the NF-κB pathway in IEC-6 cells [33]. Data from other investigators have demonstrated that CORM-2 can effectively inhibit activation of NF-κB pathway [34], [35]. Therefore, inhibition of the NF-κB pathway may be the mechanism by which CORM-2 down-regulates TNF-α and IL-1β. Since NF-κB, MAPK and MLCK are on the common pathways of LPS, TNFα and IL-1β, it is difficult to dissect the roles of LPS-induced TNFα and IL-1β in LPS-induced permeability changes. However, the resultant elevation of TNFα and IL-1β may cause a second wave of signaling and further increase the permeability of the cell monolayer. CORM-2 regulating NF-κB pathway will be expected to reduce the effects of LPS, TNFα and IL-1β. The latters will be investigated in our future studies.

Tight junctions seal the gap between adjacent epithelial cells, and prevent the passive diffusion of small molecules across the para-cellular space. Tight junction integrity plays a pivotal role in regulating the permeability of the cell monolayer. The tight junction complex consists of a series of tight junction proteins, including the trans-membrane protein occludin and intracellular plaque protein ZO-1 [36]. Previous studies have reported that LPS could down-regulate the expression of tight junction proteins and this is accompanied by an increase in permeability of the cell monolayer [37]. It has been confirmed by our study that LPS significantly down-regulates occludin, ZO-1 and claudin-1/4, and that CORM-2 can reverse this down-regulation. Our data suggest that CORM-2 may enhance the barrier function of the cell monolayer partly through up-regulating expression of tight junction proteins.

It has been demonstrated that the TJ complex associates tightly with the cytoskeleton in cells. When the cytoskeleton contracts, the TJ complex dissociates and the permeability of the cell monolayer is increased. Myosin light chain (MLC) is part of the cytoskeleton and its phosphorylation levels play a crucial role in maintenance of the function of epithelial barrier. Increase in MLC phosphorylation levels can induce the cytoskeleton to contract as well increasing the permeability of the cell monolayer [38], [39]. In our work, we observed a remarkable increase in phosphorylation levels of MLC after LPS challenge, indicating that the MLC pathway may take participate in the barrier dysfunction. Consistent with our results, an *in vivo* study also shows that LPS can increase MLC phosphorylation levels in the rat intestine and this is accompanied by disruption of the intestinal epithelial barrier [40]. In our work, we found that CORM-2 inhibited the phosphorylation of MLC. This data suggests that the MLC pathway plays a role in the CORM-2-protection of the cell monolayer barrier. However, there are two main upstream pathways, MLC and Rho kinase, which regulate the phosphorylation of MLC. Our work has not revealed which one is involved in the process [41] and this needs further investigation.

## Conclusion

In summary, the present study demonstrates that CORM-2, as a novel CO-releasing molecule, possesses an ability to protect the barrier function of intestinal epithelial cells. Inhibition of inflammatory cytokines release, restoration of TJ proteins and suppression of MLC phosphorylation levels are among the protective effects of CORM-2. Further studies are required to reveal more detailed molecular mechanisms. Our study raises a possibility that CO-releasing molecules can be developed as agents for protecting gut barrier function in inflammatory diseases.

## Supporting Information

Figure S1 **Effect of CORM-2 on the permeability of LPS-treated Caco2 cell monolayers.** Caco2 cells were grown for 21 days in transwells to achieve full differentiation. CORM-2 or inactivated CORM-2 (iCORM-2) (100 µM) was added and incubated for 1 h. The cells were then treated with 800 µg/mL LPS for 24 h. (A) Mean±SD of TER, with the UT value set 100% of 3 independent experiments are shown. (B) Permeability of FITC-dextran across the cell monolayer, with the UT value set as 100%. Results are the mean±SD of 3 independent experiments. (C) Permeability of HRP across the cell monolayer, with the value of untreated control (UT) set as 100%. Results are presented as mean±SD from 3 independent experiments. ANOVA test, *p<0.05 as compared to untreated control (UT) group,^ #^ *p*<0.05 as compared to the LPS group.
(TIF)

Figure S2 **Effect of CORM-2 on the permeability of LPS-treated T84 cell monolayer.** T84 cells were grown for 7 days in transwells. CORM-2 or inactivated CORM-2 (iCORM-2) (100 µM) was added and incubated for 1 h. The cells were then treated with 500 µg/mL LPS for 24 h. (A) Mean±SD of TER, with the UT value set 100% of 3 independent experiments are shown. (B) Permeability of FITC-dextran across the cell monolayer, with the UT value set as 100%. Results are the mean±SD of 3 independent experiments. (C) Permeability of HRP across the cell monolayer, with the value of untreated control (UT) set as 100%. Results are presented as mean±SD from 3 independent experiments. ANOVA test, *p<0.05 as compared to untreated control (UT) group, ^#^ *p*<0.05 as compared to the LPS group.
(TIF)

Figure S3 **Effect of CORM-2 on TJ protein expression in LPS-treated T84 cells.** T84 cells were pretreated with 100 µM CORM-2 or iCORM-2 for 1 h and the cells were then stimulated with 500 µg/mL LPS for 24 h. The cells were washed with PBS and lysed in clear lysis buffer. The protein concentration was determined and proteins subjected to Western blotting (Methods). (A) Representative Western blots are shown for claudin-1, claudin-4, occluding and ZO-1 with β-actin used as a loading control. The relative ratios (mean±SD) of claudin-1/β-actin (B), claudin-4/β-actin (C), occludin/β-actin (D) and ZO-1/β-actin (E) are calculated based on the densities of bands on Western blots from 3 independent experiments. ANOVA test, *p<0.05 as compared to untreated control (UT) group, ^#^ *p*<0.05 as compared to the LPS group.
(TIF)

Materials and Methods S1 **Supporting Materials and Methods.**
(DOCX)
