# Supplementary figures and images for: Protective Effects of Carbon Monoxide-Releasing Molecule-2 on the Barrier Function of Intestinal Epithelial Cells
Source: PLoS One. 2014 Aug 7;9(8):e104032. doi: 10.1371/journal.pone.0104032 (PMC4125175; doi:10.1371/journal.pone.0104032)

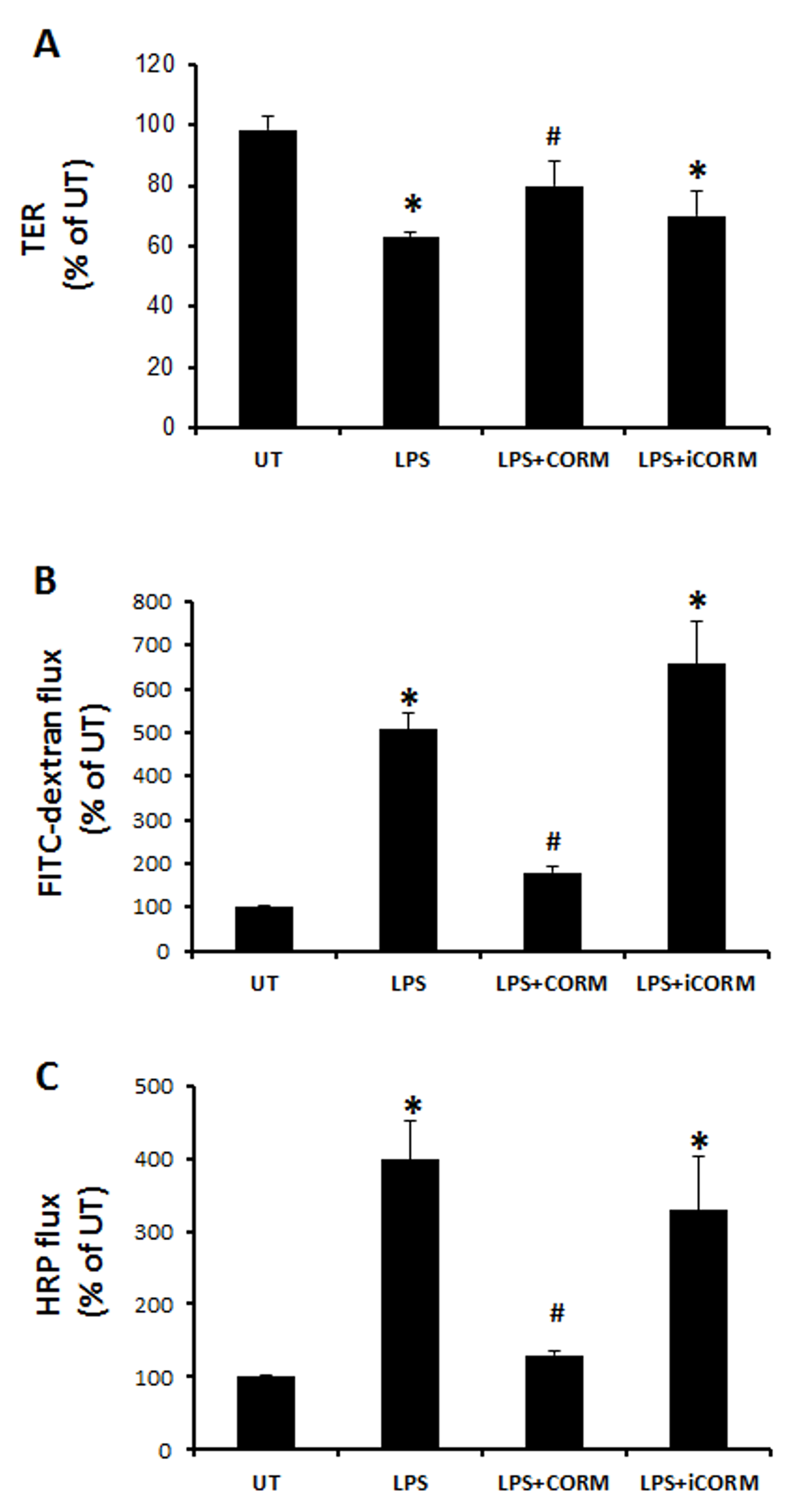

Supplement: Figure S1 — Effect of CORM-2 on the permeability of LPS-treated Caco2 cell monolayers. Caco2 cells were grown for 21 days in transwells to achieve full differentiation. CORM-2 or inactivated CORM-2 (iCORM-2) (100 µM) was added and incubated for 1 h. The cells were then treated with 800 µg/mL LPS for 24 h. (A) Mean±SD of TER, with the UT value set 100% of 3 independent experiments are shown. (B) Permeability of FITC-dextran across the cell monolayer, with the UT value set as 100%. Results are the mean±SD of 3 independent experiments. (C) Permeability of HRP across the cell monolayer, with the value of untreated control (UT) set as 100%. Results are presented as mean±SD from 3 independent experiments. ANOVA test, *p<0.05 as compared to untreated control (UT) group, # p<0.05 as compared to the LPS group. (TIF) [file pone.0104032.s001.tif]

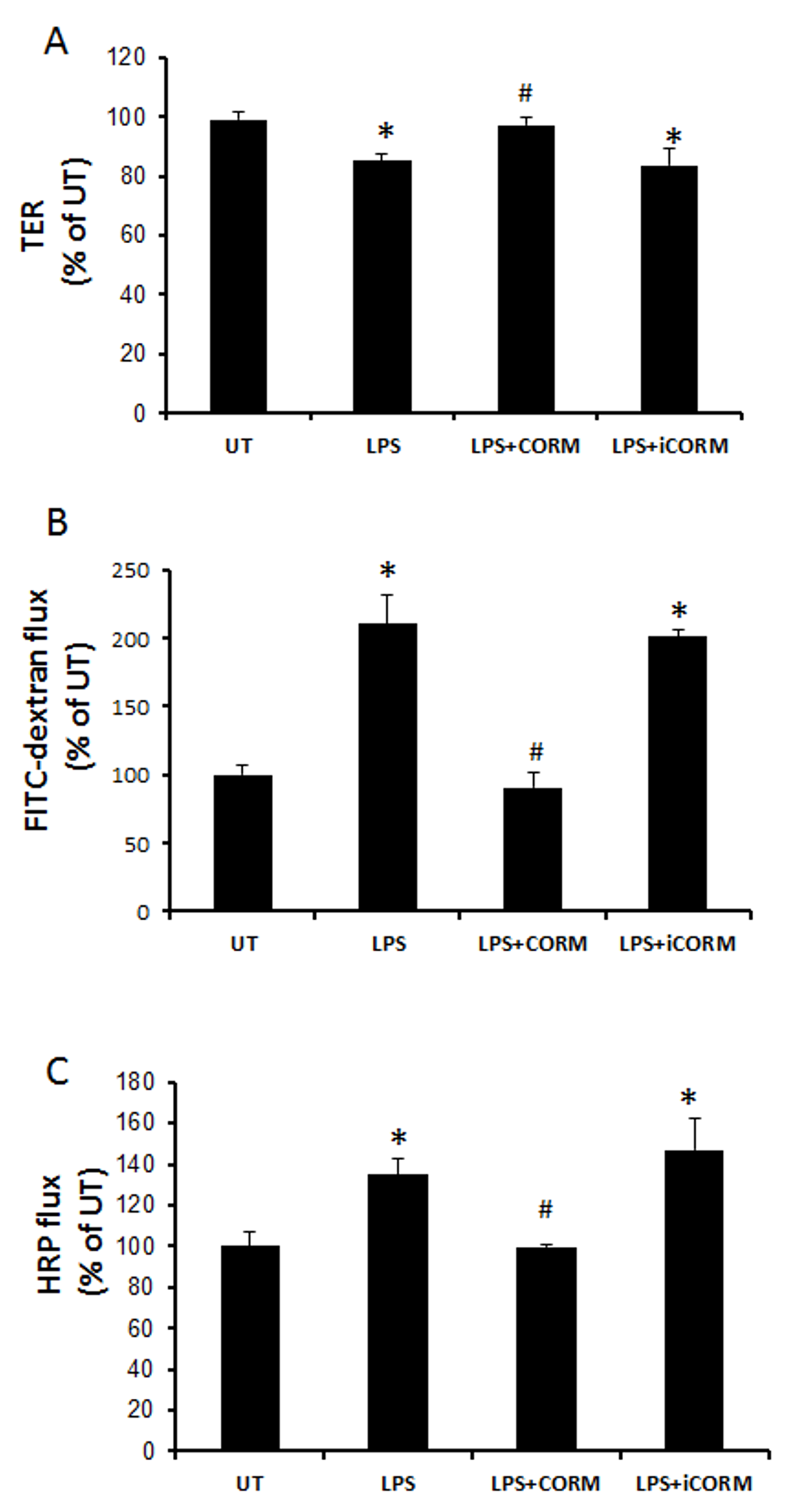

Supplement: Figure S2 — Effect of CORM-2 on the permeability of LPS-treated T84 cell monolayer. T84 cells were grown for 7 days in transwells. CORM-2 or inactivated CORM-2 (iCORM-2) (100 µM) was added and incubated for 1 h. The cells were then treated with 500 µg/mL LPS for 24 h. (A) Mean±SD of TER, with the UT value set 100% of 3 independent experiments are shown. (B) Permeability of FITC-dextran across the cell monolayer, with the UT value set as 100%. Results are the mean±SD of 3 independent experiments. (C) Permeability of HRP across the cell monolayer, with the value of untreated control (UT) set as 100%. Results are presented as mean±SD from 3 independent experiments. ANOVA test, *p<0.05 as compared to untreated control (UT) group, # p<0.05 as compared to the LPS group. (TIF) [file pone.0104032.s002.tif]

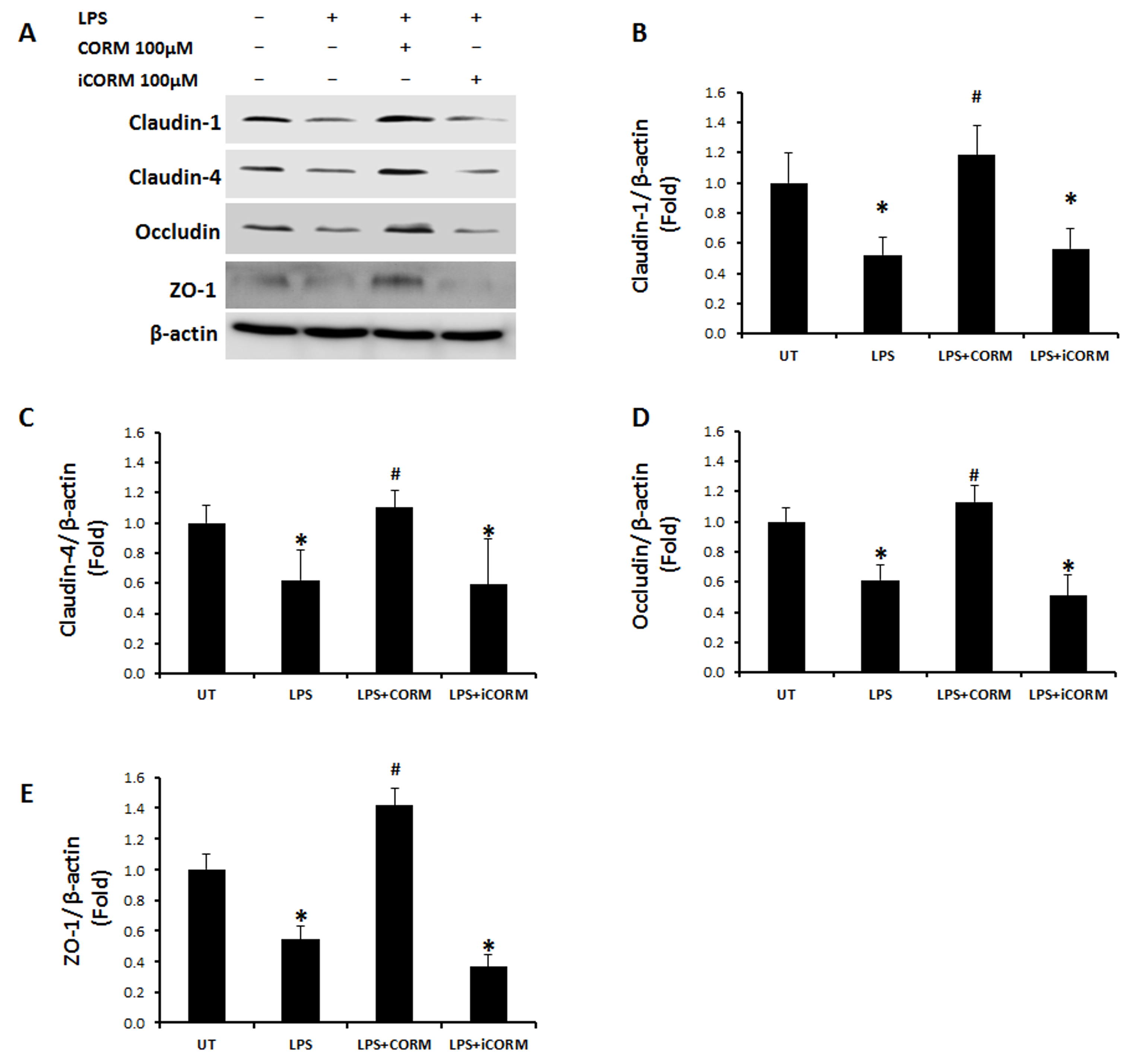

Supplement: Figure S3 — Effect of CORM-2 on TJ protein expression in LPS-treated T84 cells. T84 cells were pretreated with 100 µM CORM-2 or iCORM-2 for 1 h and the cells were then stimulated with 500 µg/mL LPS for 24 h. The cells were washed with PBS and lysed in clear lysis buffer. The protein concentration was determined and proteins subjected to Western blotting (Methods). (A) Representative Western blots are shown for claudin-1, claudin-4, occluding and ZO-1 with β-actin used as a loading control. The relative ratios (mean±SD) of claudin-1/β-actin (B), claudin-4/β-actin (C), occludin/β-actin (D) and ZO-1/β-actin (E) are calculated based on the densities of bands on Western blots from 3 independent experiments. ANOVA test, *p<0.05 as compared to untreated control (UT) group, # p<0.05 as compared to the LPS group. (TIF) [file pone.0104032.s003.tif]
