# Supplementary material for: Protective Effects of Carbon Monoxide-Releasing Molecule-2 on the Barrier Function of Intestinal Epithelial Cells
Source: PLoS One. 2014 Aug 7;9(8):e104032. doi: 10.1371/journal.pone.0104032 (PMC4125175; doi:10.1371/journal.pone.0104032)
Supplement: Materials and Methods S1 — Supporting Materials and Methods. (DOCX) [file pone.0104032.s004.docx]

**Supporting information:**

**Protective effects of** **carbon monoxide-releasing molecule-2 on the barrier function of intestinal epithelial cells**

**Materials and Methods**

**Chemicals and Reagents**

Anti-claudin-1 was purchased from Abcam. Anti-claudin-4, FITC-labeled dextran (FD-40), horseradish peroxidase (HRP), CORM-2 and LPS (*Escherichia coli* 055:B5) were purchased from Sigma-Aldrich.

**Cell culture**

Caco2 and T84 epithelial cell lines were generously provided by Professor Barry Campbell (University of Liverpool). Caco2 cells were grown using Dulbecco's modified Eagle's medium (Sigma-Aldrich) supplemented with 10% (v/v) fetal bovine serum (FBS, Gibco) and 2 mM L-glutamine. T84 cells were cultured using DMEM/F-12 medium (Gibco) supplemented with 10% (v/v) FBS and 2 mM L-Glutamine. Both cells were cultured at 37^o^C in a humidified atmosphere with 5% (v/v) CO2.

**Determination of trans-epithelial electrical resistance (TER) permeability of the Caco2 and T84 epithelial monolayer**

TER values of Caco2 and T84 cell monolayer were measured with a Millipore electrical resistance system (ERS-2; Millipore), and calculated as Ω/cm2. The cells were seeded on inserts (0.4 μM pore size; Millipore) in 24-well Transwell chambers at a density of 7.5 X 10^4^ /cm^2^. Caco2 cells were grown for 21 days to achieve full differentiation, while T84 cells were cultured for 7 days to achieve well-differentiated monolayer before experiments were performed. Only Transwells with TER values≥ 800 Ω/cm2 were used for experiments as they reflect intact Caco2 and T84 epithelial monolayer. Trans-epithelial permeability for macromolecular tracers was measured with FITC-labeled dextran (FD-40, Sigma) and HRP (44 kDa). After CORM-2 (100 µM) treatment, cells were stimulated with LPS (500 µg/mL for T84 cells) and (800 µg/mL for Caco2 cells) for 24 h. Then the medium in the bottom well was replaced with 0.5 mL DMEM, whilst medium in the upper well was replaced with 0.1 mL DMEM containing FITC-dextran at 10 mg/mL and HRP (0.5 µM). After a 6 h incubation, the amount of FITC-dextran present in the bottom well was measured with a microplate reader, while HRP was detected enzymatically using TMB substrate for horseradish peroxidase.

**Western blotting**

As described in the methods section. Claudin -1 and claudin-4 were used at a concentration of (1:2000).

**Figure S1. Effect of CORM-2 on the permeability of LPS-treated Caco2 cell monolayers.** Caco2 cells were grown for 21 days in Transwells to achieve full differentiation. CORM-2 or inactivated CORM-2 (iCORM-2) (100 µM) was added and incubated for 1 h. The cells were then treated with 800 μg/mL LPS for 24 h. (A) Means±SD of TER, with the UT value set 100% of 3 independent experiments are shown. (B) Permeability of FITC-dextran across the cell monolayer, with the UT value set as 100%. Results are the means±SD of 3 independent experiments. (C) Permeability of HRP across the cell monolayer, with the value of untreated control (UT) set as 100%. Results are presented as means±SD from 3 independent experiments. ANOVA test, **p*<0.05 as compared to untreated control (UT) group, # *p*<0.05 as compared to the LPS group.

**Figure S2. Effect of CORM-2 on the permeability of LPS-treated T84 cell monolayer.** T84 cells were grown for 7 days in transwells. CORM-2 or inactivated CORM-2 (iCORM-2) (100 µM) was added and incubated for 1 h. The cells were then treated with 500 μg/mL LPS for 24 h. (A) Means±SD of TER, with the UT value set 100% of 3 independent experiments are shown. (B) Permeability of FITC-dextran across the cell monolayer, with the UT value set as 100%. Results are the means±SD of 3 independent experiments. (C) Permeability of HRP across the cell monolayer, with the value of untreated control (UT) set as 100%. Results are presented as means±SD from 3 independent experiments. ANOVA test, **p*<0.05 as compared to untreated control (UT) group, # *p*<0.05 as compared to the LPS group.

**Figure S3. Effect of CORM-2 on TJ protein expression in LPS-treated T84 cells.** T84 cells were pretreated with 100 μM CORM-2 or iCORM-2 for 1 h and the cells were then stimulated with 500 µg/mL LPS for 24 h. The cells were washed with PBS and lysed in clear lysis buffer. The protein concentration was determined and proteins subjected to Western blotting (Methods). (A) Representative Western blots are shown for claudin-1, claudin-4, occluding and ZO-1 with β-actin used as a loading control. The relative ratios (means±SD) of claudin-1/β-actin (B), claudin-4/β-actin (C), occludin/β-actin (D) and ZO-1/β-actin (E) are calculated based on the densities of bands on Western blots from 3 independent experiments. ANOVA test,**p*<0.05 as compared to untreated control (UT) group, # *p*<0.05 as compared to the LPS group.

.
